# Low health literacy predicts decline in physical function among older adults: findings from the LitCog cohort study

**DOI:** 10.1136/jech-2014-204915

**Published:** 2015-01-08

**Authors:** Samuel G Smith, Rachel O'Conor, Laura M Curtis, Katie Waite, Ian J Deary, Michael Paasche-Orlow, Michael S Wolf

**Affiliations:** 1 Health Literacy and Learning Program, Division of General Internal Medicine and Geriatrics, Northwestern University, Chicago, Illinois, USA; 2 Wolfson Institute of Preventive Medicine, Queen Mary University of London, London, UK; 3 Department of Medicine, Boston University School of Medicine, Boston, Massachusetts, USA; 4 Department of Psychology, Centre for Cognitive Ageing and Cognitive Epidemiology, The University of Edinburgh, Edinburgh, UK; 5 Department of Learning Sciences, Northwestern University, Evanston, Illinois, USA

**Keywords:** AGEING, LONGITUDINAL STUDIES, Cohort studies, PHYSICAL FUNCTION, PSYCHOLOGY

## Abstract

**Background:**

Limited health literacy is associated with worse physical function in cross-sectional studies. We aimed to determine if health literacy is a risk factor for decline in physical function among older adults.

**Methods:**

A longitudinal cohort of 529 community-dwelling American adults aged 55–74 years were recruited from an academic general internal medicine clinic and federally qualified health centres in 2008–2011. Health literacy (Newest Vital Sign), age, gender, race, education, chronic conditions, body mass index, alcohol consumption, smoking status and exercise frequency were included in multivariable analyses. The 10-item PROMIS (Patient-Reported Outcomes Measurement Information System) physical function scale was assessed at baseline and follow-up (mean=3.2 years, SD=0.39).

**Results:**

Nearly half of the sample (48.2%) had either marginal (25.5%) or low health literacy (22.7%). Average physical function at baseline was 83.2 (SD=16.6) of 100, and health literacy was associated with poorer baseline physical function in multivariable analysis (p=0.004). At follow-up, physical function declined to 81.9 (SD=17.3; p=0.006) and 20.5% experienced a meaningful decline (>0.5 SD of baseline score). In multivariable analyses, participants with marginal (OR 2.62; 95%CI 1.38 to 4.95; p=0.003) and low (OR 2.57; 95%CI 1.22 to 5.44; p=0.013) health literacy were more likely to experience meaningful decline in physical function than the adequate health literacy group. Entering cognitive abilities to these models did not substantially attenuate effect sizes. Health literacy attenuated the relationship between black race and decline in physical function by 32.6%.

**Conclusions:**

Lower health literacy increases the risk of exhibiting faster physical decline over time among older adults. Strategies that reduce literacy disparities should be designed and evaluated.

## Introduction

In 2012, 19% of the US population was aged 60 years and over.^1^ This is projected to rise to 23% by 2020 and 27% by the middle of the century,^2^ underscoring the need to promote healthy aging as a public health priority.^3^ Physical function is an important outcome to monitor, and has been associated with multiple risk factors including risk of falling,^4^ cognitive decline^5^ and all-cause mortality.^6^ Reviews suggest a trend for higher levels of physical function in recent years,^7^ ^8^ but significant disparities among population subgroups are evident.^8^ ^9^ Further investigation is required to identify mechanisms through which decline in functional health can be slowed or prevented among older adults.

Health literacy is a potential risk factor for poor physical function. The Institute of Medicine define the construct as ‘the degree to which individuals have the capacity to obtain, process, and understand basic health information and services needed to make appropriate health decisions’.^10^ Theoretical frameworks and empirical research provide support for a hypothesised relationship between health literacy and decline in physical function.^11^ For example, health literacy is a risk factor for health outcomes associated with physical function such as poor medication adherence,^12^ health behaviours^13^ and lower uptake of preventive services (eg, cancer screening and vaccinations).^14^ Estimates from a nationally representative study suggest between one-third and one-half of adults have low health literacy, with lower socioeconomic status and minority groups over-represented.^15^ The prevalence of low health literacy is markedly higher among older adults.^16^

Cross-sectional studies have shown associations between health literacy and physical function among older adults.^17–19^ In a sample of 2923 older Medicare managed care enrollees, respondents with low or marginal health literacy skills had worse physical function, experienced more difficulties with activities of daily living and reported more limitations in physical activity.^20^ There is also cross-sectional evidence to suggest the relationship between health literacy and health outcomes is at least partially explained by cognitive ability.^18^ ^21^ ^22^ Longitudinal studies are needed to test whether decline in physical function over time is more pronounced among people with lower health literacy, and whether this relationship can be explained by cognitive function.

This study investigated the association between health literacy and a meaningful decline in physical function among a cohort of community-dwelling older American adults. It was hypothesised that people with marginal and low health literacy would be more likely to report worse physical function at baseline, and experience a higher likelihood of meaningful decline in physical function than those with adequate health literacy at follow-up.

## Methods

### Sample

The Health Literacy and Cognitive Function among Older Adults cohort (also known as ‘LitCog’) were recruited from one academic general internal medicine ambulatory care clinic and five federally qualified health centres in Chicago, Illinois.^21^ Recruitment took place from August 2008 through October 2011. English-speaking adults between the ages of 55–74 years (n=3176) were identified, approached by telephone and 1904 were eligible. Screening led to 244 exclusions due to limited English proficiency, severe cognitive or hearing impairment or not being associated with a clinic physician (ie, had less than two visits in the previous 2 years). Cognitive impairment was assessed using a brief six-item screener.^23^ A total of 794 people refused, 14 were deceased and 20 were eligible but had scheduling conflicts. The final sample included 828 participants for a cooperation rate of 51%. At follow-up, 529 (64%) were retained.

### Procedure

At baseline, participants completed two in-person structured interviews 7–10 days apart. Our a priori protocol was to contact participants after 2.5 years. However, to maximise retention among ‘hard-to-reach’ groups, participation was permitted between 2 and 5 years postbaseline interview. The average time to follow-up was 3.2 years (SD=0.39). A similar battery was administered at the follow-up interview. All participants gave informed consent prior to the study, Northwestern University's Institutional Review Board approved the study (STU00026255), and the principles embodied by the Declaration of Helsinki were adhered to.

### Measures

#### Health literacy

The Newest Vital Sign (NVS) is an objective assessment of health literacy.^24^ A trained interviewer administers six open-ended questions which can be answered using information on a nutritional label. Numeracy and literacy skills are required for successful completion. Score range is 0–6, with 1 point allocated for each correct answer. Scores are classified in terms of likelihood of limited literacy (0–1: likely limited; 2–3: possibly limited; 4–6: adequate).^24^

The Test of Functional Health Literacy in Adults (TOFHLA) is an objective health literacy measure.^25^ It is composed of a numeracy (17 items) and a literacy section (50 items). The numeracy section assesses comprehension of actual information materials that a patient might encounter (eg, a prescription label, an appointment slip, a chart describing eligibility for financial aid, an example results from a medical test). The reading assessment uses the cloze procedure whereby every fifth to seventh word of a text is missing, and the participant selects the most appropriate missing word from a list of four. Scores are classified as low (0–59), marginal (60–74) or adequate (75–100).^25^

The Rapid Estimate of Adult Literacy in Medicine (REALM)^26^ is a word-recognition test containing 66 health-related words. Participants are asked to read through this list, which increases in difficulty. One point is awarded per correct pronunciation, and scores are classified as low (0–44), marginal (45–60) or adequate (61–66).^26^ For clarity, health literacy categories for all three measures are herein referred to as low, marginal or adequate.

#### Physical function

The 10-item short form of the PROMIS (Patient-Reported Outcomes Measurement Information System) physical function scale was used.^27^ The scale assesses the ability to perform everyday physical activities such as dressing and bathing. Scores are transformed to range from 0 to 100, with higher scores indicating better physical function. Five questions are phrased, ‘Does your health now limit you in doing…’ and example activities included walking more than a mile and climbing one flight of stairs. Response options were, ‘Not at all’, ‘very little’, ‘somewhat’, ‘quite a lot’, ‘cannot do’. A further five questions are phrased, ‘Tell me if you are able to…’ and example tasks included ‘shampoo your hair’ and ‘get on and off the toilet’. Response options for these items were, ‘without any difficulty’, ‘with a little difficulty’, ‘with some difficulty’, ‘with much difficulty’ and ‘unable to do’. The scale was reliable in the baseline and follow-up samples (α=0.90; and α=0.89 respectively).

### Cognitive function

Sixteen cognitive tests were used to assess six cognitive domains that reflect fluid cognitive abilities (processing speed, working memory, inductive reasoning, long-term memory, prospective memory) and crystallised abilities (verbal ability). Fluid abilities represent cognitive traits which facilitate information processing where prior general knowledge is not useful. Crystallised abilities reflect general background knowledge stored in long-term memory. Factor analysis was used to derive latent traits for fluid and crystallised skills. The specific tests used, their source, and a brief description can be found in the baseline LitCog report^21^ or in the online supplementary appendix.

#### Participant characteristics

Measures of age, gender, race (white, black, other), education (high school or less, some college or technical school, college graduate, graduate degree), and the number of self-reported chronic conditions was recorded (diabetes, pulmonary oedema, chronic obstructive pulmonary disease, coronary artery disease, asthma, hypertension, arthritis, cancer, depression). The number of chronic conditions was categorised as 0, 1 and 2 or more. Smoking was self-reported using the item, ‘How would you describe your cigarette smoking habits?’ (current smoker, former smoker, non-smoker). Self-reported exercise frequency was recorded using the item, ‘In an average week, how many times do you engage in physical activity for at least 20 min? Specifically, exercise or work which is hard enough to make you breathe heavier and your heart beat faster?’ (≥4 times per week, 3 times per week, 1 or 2 times per week, <1 time per week). Participants were classified as being active ≥4 or <4 times per week. Alcohol intake was assessed using the item, ‘In an average week, how often do you drink alcohol?’ (≥4 drinks per week, 3 drinks per week, 1 or 2 drinks per week, <1 drink per week). Responses were dichotomised (<1 or ≥1 drink per week). Body mass index (BMI) was calculated from self-reported height and weight. Participants were classified as normal weight (<25 kg/m^2^), overweight (≥25–29.9 kg/m^2^) and obese (≥30 kg/m^2^).

### Analysis plan

Participant characteristics were described, and differences between those who were retained and not retained were analysed using χ^2^ analyses. Differences between participant characteristics and baseline physical function were analysed using analysis of variance (ANOVA) and then adjusted using analysis of covariance (ANCOVA). Partial eta^2^ effect sizes were reported for ANOVA and ANCOVA analyses. Differences in physical function between baseline and follow-up were assessed using within-subjects t tests.

Crystallised and fluid cognitive ability scores were created to reduce cognitive skills to one measure per category and to avoid multicollinearity in regression models. Univariate imputation sampling methods were used to estimate missing values (n=98) on cognitive measures by regressing each variable on age and variables from the same cognitive category in a bootstrapped sample of non-missing observations. Crystallised and fluid cognitive ability scores were calculated by estimating a single-factor score with maximum likelihood estimation.^21^

The effect size method was used to calculate decline in physical function.^28^ This method calculates a standardised measure of change over time. The difference between baseline and follow-up scores is divided by the SD at baseline. The resulting effect size represents change in terms of the number of baseline SD. A medium effect size (0.50) represented a meaningful decline in physical function.^28^ In this sample, a medium effect size corresponded to an absolute decline on the 0–100 scale of approximately 8 points. To investigate the predictors of decline, χ^2^ and multivariable logistic regression analyses were performed. The multivariable model contained health literacy (NVS), age, gender, race, education, smoking status, exercise frequency, alcohol, BMI, chronic conditions, time since baseline interview and baseline physical function. The binary outcome was ‘meaningful decline’ or ‘no meaningful decline’ in physical function. In subsequent analyses, cognitive abilities were entered into multivariable logistic regression models to observe potential attenuation of the relationship between health literacy and decline in physical function. Step-wise logistic regression investigated whether health literacy attenuated the relationship between race and functional decline. The NVS was the main health literacy measure used, but analyses comparing baseline differences in physical function, and decline after follow-up were repeated using the TOFHLA and REALM (online supplementary appendix). With the exception of the cognitive ability measures discussed above and BMI (n=14, 2.7%), missing data were less than 1% for independent and dependent variables. Pairwise deletion was used. The type 1 error rate was set at p<0.05. Analyses were performed in SPSS V.22.

## Results

### Participant characteristics

Of the 828 participants who took part in LitCog at baseline, 826 (99.8%) provided data on physical function. At follow-up, 529 (63.9%) participants were interviewed and 100% provided physical function data. The average time to follow-up was 3.2 years (SD=0.39). The following factors were associated with higher retention at follow-up: white race (p<0.001), non-smoking status (p=0.001), drinking one or more alcoholic drinks per week (p=0.001), fewer chronic conditions (p=0.006), higher health literacy (NVS, p=0.001), higher education (p<0.001) and better baseline physical function (p=0.001).

The average age of the sample at baseline was 63 years (SD=5.42), ranging from 55 to 74. At follow-up the average age was 66 years (SD=5.4), ranging from 58 to 79. Participants were mostly female (68.6%), white (56.4%) or black (37%), and had a graduate degree (36.3%, table 1). Most (83.5%) had at least one chronic condition. As recorded at baseline, the majority of participants were non-smokers (89.4%), exercised <4 times per week (59.6%), and drank <1 alcoholic drink per week (58.9%). BMI categories were evenly distributed between normal weight (32.6%), overweight (31.3%) and obese (36.1%). Over half of the sample had adequate health literacy as assessed by the NVS (51.8%), with 22.7% and 25.5% classified as marginal and low health literacy, respectively.

**Table 1:** Participant characteristics and baseline differences in physical function

		Physical function		Adjusted p value
	n (%)	Mean (SD)	Unadjusted p value	
Age			0.985	0.641
55–60	195 (36.9)	83.4 (18.7)		
61–65	150 (28.4)	82.8 (16.1)		
66–70	122 (23.1)	83.4 (15.1)		
71–74	62 (11.7)	83.3 (13.6)		
Gender			0.214	0.342
Male	166 (31.4)	84.5 (16.5)		
Female	363 (68.6)	82.6 (16.6)		
Race			<0.001	0.653
White	297 (56.4)	87.3 (13.5)		
Black	195 (37.0)	77.1 (19.0)		
Other	35 (6.6)	82.4 (15.8)		
Education			<0.001	0.179
≤High school	116 (21.9)	76.7 (17.9)		
Some college or tech	112 (21.2)	78.3 (18.5)		
College graduate	109 (20.6)	85.4 (15.9)		
Graduate degree	192 (36.3)	88.8 (12.4)		
Smoking status			<0.001	0.128
Current smoker	56 (10.6)	74.9 (19.5)		
Former smoker	171 (32.3)	83.3 (15.4)		
Never smoked	302 (57.1)	84.7 (16.3)		
Exercise frequency			<0.001	0.021
≥4 times per week	213 (40.4)	87.5 (15.2)		
<4 times per week	314 (59.6)	80.3 (16.9)		
Alcohol			<0.001	0.612
<1 per week	311 (58.8)	80.3 (17.3)		
≥1 per week	218 (41.2)	87.3 (14.6)		
BMI			<0.001	0.001
Normal weight	168 (32.6)	88.7 (14.2)		
Overweight	161 (31.3)	84.5 (16.0)		
Obese	186 (36.1)	78.0 (17.3)		
Chronic conditions			<0.001	<0.001
0	87 (16.4)	93.8 (10.1)		
1	163 (30.8)	88.8 (12.8)		
2+	279 (52.7)	76.7 (17.3)		
Health literacy (NVS)			<0.001	0.004
Low	135 (25.5)	75.5 (18.1)		
Marginal	120 (22.7)	82.2 (18.1)		
Adequate	274 (51.8)	87.5 (13.5)		

Adjusted p values were multivariable analyses controlling for all characteristics in the table.

BMI, body mass index; NVS, Newest Vital Sign.

### Baseline physical function

The average level of physical function at baseline was 83.2 (SD=16.6). In analyses adjusted for participant characteristics, there were baseline differences in physical function by exercise frequency (p=0.021, ηρ²=0.01), BMI (p=0.001, ηρ²=0.02), chronic conditions (p<0.001, ηρ²=0.10) and health literacy (NVS, p=0.004, ηρ²=0.02; table 1).

We repeated these analyses using the TOFHLA and REALM. In comparison with the NVS, there were fewer individuals with the lowest level of health literacy using these measures (NVS, 25.5%; TOFHLA, 9.4% and REALM, 6.6%). There were univariable differences between the TOFHLA and REALM groups in baseline physical function (both p<0.001), but these differences were reduced to non-significance in multivariable models (online supplementary appendix).

### Predicting meaningful decline in physical function

At follow-up, the average level of physical function was 81.9 (SD=17.3) and this was significantly lower than baseline levels (p=0.006). Approximately one-fifth (20.5%) of the sample had a meaningful decline in physical function between baseline and follow-up. Among these patients, the average decline in physical function was −17.5 (SD=8.4).

To investigate the predictors of meaningful decline in physical function, participants who had the same or improved scores at follow-up were combined and compared with those who met our threshold for meaningful decline (table 2). Multivariable logistic regression analyses controlling for participant characteristics, health behaviours, time since baseline interview and baseline physical function scores were performed. In comparison with those who had adequate health literacy skills on the NVS measure, the marginal (OR 2.62; 95% CI 1.38 to 4.95; p=0.003) and low (OR 2.57; 95% CI 1.22 to 5.44; p=0.011) groups were more likely to experience meaningful decline in physical function. Participants with two or more chronic conditions were more likely to report a meaningful decline in physical function than those with no chronic conditions (OR 2.96; 95% CI 1.29 to 6.81; p=0.011). There was no effect for having one chronic condition (OR 1.44; 95% CI=0.61 to 3.41; p=0.409). Compared with normal weight participants, those who were overweight (OR 2.23; 95% CI 1.17 to 4.25; p=0.015) or obese (OR 2.10; 95% CI 1.10 to 4.01; p=0.025) were more likely to experience decline in physical function. There was no effect for age, gender, race, education, smoking status, exercise frequency, alcohol consumption and time since baseline interview (p>0.05). We repeated these analyses using the TOFHLA and REALM. There were univariable differences in physical function decline across both measures (p=0.003 and p<0.001, respectively), but these differences were not significant in multivariable models (online supplementary appendix).

**Table 2:** Univariable and multivariable analyses predicting physical function decline

		Physical function			
	No decline (%)	Decline (%)	χ^2^ p Value	OR (95%CI)	p Value
Age			0.037		
55–60	80.0	20.0		Ref	Ref
61–65	85.9	14.1		0.62 (0.33 to 1.18)	0.144
66–70	76.2	23.8		1.14 (0.62 to 2.12)	0.669
71–74	69.4	30.6		1.66 (0.78 to 3.54)	0.189
Gender			0.358		
Male	81.9	18.1		0.83 (0.48 to 1.44)	0.508
Female	78.5	21.5		Ref	Ref
Race			<0.001		
White	85.9	14.1		Ref	Ref
Black	70.1	29.9		1.37 (0.72 to 2.60)	0.334
Other	77.1	22.9		1.21 (0.46 to 3.23)	0.699
Education			0.007		
≤High school	69.6	30.4		1.05 (0.47 to 2.33)	0.908
Some college or tech	77.7	22.3		1.07 (0.52 to 2.21)	0.863
College graduate	80.7	19.3		1.18 (0.59 to 2.35)	0.650
Graduate degree	85.9	14.1		Ref	Ref
Smoking status			0.067		
Current smoker	80.4	19.6		0.85 (0.37 to 1.98)	0.714
Former smoker	81.9	18.1		0.58 (0.25 to 1.35)	0.209
Never smoked	67.9	32.1		Ref	Ref
Exercise frequency			0.190		
≥4 times per week	82.6	17.4		Ref	Ref
<4 times per week	78.0	22.0		0.91 (0.55 to 1.51)	0.721
Alcohol			0.001		
<1 per week	74.8	25.2		Ref	Ref
≥1 per week	86.2	13.8		1.36 (0.77 to 2.38)	0.288
BMI			0.033		
Normal weight	86.3	13.7		Ref	Ref
Overweight	77.6	22.4		2.23 (1.17 to 4.25)	0.015
Obese	75.7	24.3		2.10 (1.10 to 4.01)	0.025
Chronic conditions			<0.001		
0	89.7	10.3		Ref	Ref
1	85.3	14.7		1.44 (0.61 to 3.41)	0.409
2+	73.0	27.0		2.96 (1.29 to 6.81)	0.011
Health literacy			<0.001		
Low	69.4	30.6		2.57 (1.22 to 5.44)	0.013
Marginal	71.7	28.3		2.62 (1.38 to 4.95)	0.003
Adequate	88.0	12.0		Ref	Ref

Multivariable analyses were adjusted for all characteristics, as well as time since baseline interview (≤36 months, >36 months) and baseline physical function.

BMI, body mass index.

### Cognition, health literacy and decline in physical function

To identify the extent to which baseline cognition explained the relationship between health literacy assessed by the NVS and decline in physical function, a series of logistic regression models were performed (table 3). In multivariable models, fluid cognitive ability was not associated with decline in physical function (OR=0.73; 95% CI 0.48 to 1.01; p=0.132) and it only marginally attenuated the association between health literacy and physical function decline (low: OR=2.01; 95% CI 0.88 to 4.62; p=0.099; 22% attenuation; marginal: OR=2.32, 95% CI 1.18 to 4.54; p=0.014; 11% attenuation). Crystallised ability was also not associated with decline in physical function (OR=0.85; 95% CI 0.56 to 1.31; p=0.473) and its effect on the relationship between health literacy and physical function decline was small (low: OR=2.32; 95% CI 1.02 to 5.30; p=0.044; 10% attenuation; marginal: OR=2.45, 95% CI 1.25 to 4.81; p=0.009; 6% attenuation). Adding fluid abilities and crystallised abilities to the multivariable model yielded no substantial changes to these observations (table 3).

**Table 3:** Step-wise logistic regression examining the effect of HL and cognition on decline in physical function

	HL only	HL and FA	HL and CA	HL, FA and CA
Health literacy				
Low	2.57 (1.22 to 5.44)*	2.01 (0.88 to 4.62)	2.32 (1.02 to 5.30)*	2.06 (0.87 to 4.88)
Marginal	2.62 (1.38 to 4.95)**	2.32 (1.18 to 4.54)*	2.45 (1.25 to4.81)**	2.30 (1.15 to 4.58)*
Adequate	Ref	Ref	Ref	Ref
Fluid cognitive ability	–	0.73 (0.48 to 1.01)	–	0.80 (0.50 to 1.27)
Crystallised cognitive ability	–	–	0.85 (0.56 to 1.31)	0.94 (0.58 to 1.50)

These are multivariable analyses controlling for age, gender, race, education, smoking status, exercise frequency, alcohol consumption, BMI, chronic conditions, baseline physical function and time to follow-up.

*p<0.05, **p<0.01.

BMI, body mass index; CA, crystallised abilities; FA, fluid abilities; HL, health literacy.

### The mediating role of health literacy in racial disparities

The extent to which health literacy, as assessed by the NVS, mediated the association between race and decline in physical function was investigated in step-wise multivariable analyses. At step 1, black race was associated with decline in physical function (OR 2.59; 95% CI 1.65 to 4.05; p<0.001). This association was attenuated by 32.6% when adding health literacy to the model, but not to the point of non-significance (OR 1.74; 95% CI 1.03 to 2.95; p=0.038).

## Discussion

In this cohort of community-dwelling older Americans, participants with low and marginal health literacy as assessed by the NVS had poorer physical function at baseline. As hypothesised, after an average follow-up of 3 years, respondents with lower health literacy were over 2.5 times more likely to experience a clinically meaningful decline in physical function. In absolute terms, this was a decline in physical function of approximately 8 points on a 0–100 scale. These findings were maintained even after controlling for baseline physical function, participant characteristics (including chronic conditions, education and race), health behaviours and BMI. Groups with limited and marginal health literacy are more likely to experience poorer physical health generally, and they may experience the effects of aging more rapidly.

After controlling for confounding variables, there was no association between the TOFHLA or REALM and baseline physical function. This is likely to be due to the relative ease of these assessments in comparison with the NVS, resulting in smaller proportions of people being classified as having low health literacy. Low agreement between health literacy measures has previously been documented, suggesting these assessments measure different skill sets.^29^ In this sample, nearly half had either limited or marginal health literacy when assessed by the NVS (48%), compared with 26% and 21% on the TOFHLA and REALM, respectively. Identifying the differences between these measures and their optimal cut-offs for classifying low health literacy could be an important next step in understanding their relationships with health outcomes.

An emerging programme of work has demonstrated substantial attenuation of the relationship between health literacy and health outcomes when cognitive abilities are considered.^18^ ^21^ ^22^ However, there was no evidence from within the prospective data reported here that the ability to actively learn new concepts and procedures (fluid skills) or one's background knowledge and verbal ability (crystallised skills) attenuated the relationship between health literacy and physical function decline. This raises the possibility that health literacy as assessed by the NVS is making a unique contribution to decline in physical function among older adults. Future research should continue to explore these relationships, as identifying the most relevant construct to target will have important implications for designing and implementing strategies to ameliorate disparities.^30^ ^31^

These findings build on research demonstrating cross-sectional associations between physical function and health literacy among older adults.^17–20^ No other studies have investigated the association between health literacy and decline in physical function over time among older adults. A body of evidence is available linking lower health literacy with outcomes that could explain our observations.^17^ There is a moderate to strong evidence base linking limited health literacy with inappropriate medication use,^12^ risk of hospitalisation,^32^ inaccurate processing of health information,^33^ and lower uptake of preventive health services (eg, cancer screening).^14^ These cumulative effects may help explain the greater levels of physical decline among lower health literacy groups.

Clinicians treating older adults and policy makers implementing public health strategies should also be aware that a large proportion of adults have limited levels of health literacy. For example, a meta-analysis of 31 129 adults from 85 studies found a quarter had limited health literacy.^16^ However, when restricting this to studies with an average age of over 50 years, the figure rose to 38%. Education was not related to physical function in either of our multivariable models suggesting it should not be used as a marker for health literacy. Education may be a poor proxy of the skills needed by older adults to function in the healthcare system because it fails to consider lifelong learning and age-related declines in cognitive ability.^34^ While health literacy screening in clinical practice is not recommended because of a poor evidence base,^35^ heightened awareness of the prevalence of patients with low health literacy skills among clinicians may help them to meet the needs of their patients.

In exploratory analyses, health literacy attenuated the relationship between black race and decline in physical function. This supports a growing number of studies indicating that health literacy may be a key mediator of racial disparities.^36^ ^37^ It is recommend that researchers undertake similar analyses to investigate this phenomenon. This finding raises the possibility that if barriers to appropriate health self-management were reduced for low health literacy groups, there may be concomitant effects on racial disparities.

Further efforts are needed at all stages of the development, evaluation and dissemination of health literacy interventions. However, there are some promising strategies worth highlighting.^38^ In a cluster-randomised trial of a healthcare provider intervention, colorectal screening rates were increased among a sample of veterans aged 50 years, with a proportionately greater effect among lower health literacy participants.^39^ The intervention involved a 2 hour workshop on improving communication skills with low-literacy patients (small discussions and role-playing sessions), and a 1 hour session every 4–6 months providing feedback on patient uptake rates. Other interventions that have been shown to have greater effects among groups with poor basic skills include: one-to-one education sessions using the ‘teach back’ method for using medical devices;^40^ ^41^ designing health education materials so that less important information is presented last or not at all;^42^ providing visual aids such as bar charts and icon arrays when conveying numerical information;^43^ ^44^ and clarifying ambiguous wording on medication dosing instructions.^45^ ^46^ Although these strategies have shown promise, the next steps for the field should be focused on the continued development of effective and scalable interventions to ameliorate health literacy disparities.

This study has limitations. Levels of repeat participation in the study were good (64%). However, individuals were more likely to be retained at follow-up if they were white, educated and had adequate health literacy skills. They also reported fewer chronic conditions, were more likely to be non-smokers, and have higher baseline physical function. This sample should therefore be considered a healthier and less disadvantaged subsample of those that participated at baseline. Demographic data were unavailable for those who were initially identified, but did not participate. It is therefore unclear how comparable the LitCog cohort is to the pool of people from which they were selected. We were unable to record all of the chronic conditions that may affect physical function. Participants were recruited from multiple health clinics, but all were based within the same city. The average age of participants at baseline was 63 years, and 65% were aged 65 years or less at baseline. Larger declines might be expected as this cohort continues to age. Finally, all outcomes and exposure variables used in the study were self-reported, and therefore may have been prone to response biases. Objective measures of physical function are more reliable and sensitive to decline, but they were unavailable in this cohort.

In conclusion, among a cohort of community-dwelling older American adults, people with lower health literacy were more likely to experience poorer physical function at baseline and they exhibited a higher rate of meaningful functional decline over time. Health literacy also explained a substantial portion of the relationship between black race and decline in physical function. Following on from a number of cross-sectional studies, this is the first prospective study to report these associations. Clinicians and policy makers should be aware of how prevalent limited health literacy is among older people, and evidence-based strategies that ameliorate health literacy disparities should be designed, tested and incorporated into public health and clinical settings.

**What is already known on this subject**

- Associations between health literacy and physical function have been reported in cross-sectional studies. However, longitudinal studies are needed to establish whether decline in physical function is more pronounced among those with poor basic skills.

**What this study adds**

- We established that the physical effects of ageing are more pronounced among people with low health literacy, even after controlling for sociodemographic background, cognitive abilities, health behaviours and body mass index. These findings have implications for clinicians, researchers and policy makers working to promote healthy ageing.

## Supplementary Material

Web supplement
