# Supplementary material for: Low health literacy predicts decline in physical function among older adults: findings from the LitCog cohort study
Source: J Epidemiol Community Health. 2015 Jan 8;69(5):474–80. doi: 10.1136/jech-2014-204915 (PMC4413744; doi:10.1136/jech-2014-204915)
Supplement: Web supplement [file jech-2014-204915-s1.pdf]

Online Appendix

Trait	Measure	Source	Description
Fluid abilities			
Processing speed	Digital comparison	Salthouse	Compare strings of digits that are the same or different, completing as many trials as possible in a given time period.
	Pattern comparison	Salthouse & Babcock	Compare pairs of simple line drawings that are the same or different, completing as many trials in a given time period.
	Symbol digit modalities	WPS	Match symbols to appropriate digits via a legend marking designated symbol-digit pairings, completing as many matches as possible in a given time period.
Working memory	Spatial Span Length – Reverse	CANTAB	Participants are shown differently sized sets of boxes highlighted in specific orders and then must indicate the reverse order in which each set of boxes were highlighted.
	Spatial Working Memory (SWM)	CANTAB	Find tokens hidden behind boxes while clicking as few boxes as possible, keeping track of which boxes have already been searched.
	Size Judgement Span	Cherry & Park	Read lists of differing amounts of randomly ordered, size-constant items and reorder items from smallest to largest.
Inductive Reasoning	ETS Letter Sets	ETS	Participants are shown series of sets of letters and must decide which set of letters in each series does not follow the same pattern as the other sets in that series.
	Ravens Progressive Matrices	Raven	Participants are shown incomplete sets of patterns and must decide from multiple options which additional pattern would complete each set.
	Stockings of Cambridge (SOC)	CANTAB	Match one series of coloured balls sitting in stockings to another, allowing for movement of only one ball at a time and aiming to match the sets in as few moves as possible.
Long-Term Memory	Immediate Verbal Memory	CANTAB	Participants are shown a list of words and immediately asked to freely recall as many of the words as possible.
	Delayed Verbal Memory	CANTAB	Freely recall as many words as possible from a previously read list after a delay of approximately 20 minutes.
	New York Paragraph	Kluger	Read a short story and repeat back as much of the story as possible after an approximately 20 minute delay.
Prospective Memory	LitCog 3-Step Assessment	Wolf	Participants are asked at the beginning of interview to remember to complete three simple tasks throughout the interviews (writing down times of a break and interview end time, returning a visitor pass).
Crystallised abilities			
Verbal ability	AM-NART	Grober	Read aloud a list of general words and assess for correct pronunciation.
	Graded Naming Test	CANTAB	Participants are shown a series of images depicting different objects and asked to identify each object.
	Shipley Institute of Living Scale	Shipley	Participants must identify synonymous words via a multiple choice paradigm.

References

Salthouse TA. What do adult age differences in the digit symbol substitution test reflect? *J Gerontol: Psychol Sci* 1992;**47**:121–8.

Salthouse TA, Babcock RL. Decomposing adult age differences in working memory. *Dev Psychol* 1991;**27**:763–76.

Smith A. Symbol digit modalities test. Los Angeles: Western Psychological Services. 1991.

Robbins TW, James M, Owen AM, Sahakian BJ, McInnes L, Rabbitt PMA. Cambridge Neuropsychological Test Automated Battery (CANTAB): A factor analytic study of a large sample of normal elderly volunteers. *Dement*. 1994;**5**:266–81.

Cherry KE, Park DC. Individual difference and contextual variables influence spatial memory in younger and older adults. *Psychol Aging*. 1993;**8**:517–7.

Ekstrom RB, French JW, Harman HH. ETS kit of factor-referenced cognitive tests. Princeton, NJ: Educational Testing Service; 1976.

Raven JC. Standard progressive matrices: sets A, B, C, D and E. San Antonio, TX: Harcourt Assessment; 1976.

Grober E, Sliwinsk M, Korey SR. Development and validation of a model for estimating premorbid verbal intelligence in the elderly. *J Clin Exp Neuropsychol*. 1991;**13**:933–949.

Zachary RA.*Shipley Institute of Living Scale, Revised Manual*. Los Angeles, CA: Western Psychological Services, 1986; 1986.

Kluger A, Ferris SH, Golomb J, Mittelman MS, Reisberg B. Neuropsychological prediction of decline to dementia in nondemented elderly. *J Geriatr Psychiatry Neurol*. 1999;**12**:168.

Test of Functional Health Literacy in Adults (TOFHLA) and baseline differences in physical function

	Physical Function			
	n (%)	Mean (SD)	Unadjusted p value	Adjusted p value
TOFHLA			<0.001	0.135
Low	49 (9.4)	72.9		
Marginal	84 (16.1)	76.9		
Adequate	390 (74.6)	85.8		

Note: Adjusted p-values were multivariable analyses controlling for age, gender, race, education, smoking, exercise frequency, alcohol consumption, BMI, and chronic conditions

Rapid Estimate of Adult Literacy in Medicine (REALM) and baseline differences in physical function

	Physical Function			
	n (%)	Mean (SD)	Unadjusted p value	Adjusted p value
REALM			<0.001	0.941
Low	35 (6.6)	74.4		
Marginal	78 (14.7)	76.3		
Adequate	416 (78.6)	85.3		

Note: Adjusted p-values were multivariable analyses controlling for age, gender, race, education, smoking, exercise frequency, alcohol consumption, BMI, and chronic conditions

Test of Functional Health Literacy in Adults (TOFHLA) and physical function decline

	Physical function				
	No decline (%)	Decline (%)	χ^2 p value	OR (95%CI)	p value
TOFHLA			0.003		
Low	66.7	33.3		1.18 (0.51-2.77)	0.698
Marginal	71.4	28.6		0.99 (0.50-1.97)	0.971
Adequate	83.1	16.9		Ref	Ref

Note: Multivariable analyses were adjusted for age, gender, race, education, smoking, exercise frequency, alcohol consumption, BMI, and chronic conditions, time since baseline interview (≤36 months, >36 months) and baseline physical function

Rapid Estimate of Adult Literacy in Medicine (REALM) and physical function decline

	Physical function				
	No decline (%)	Decline (%)	χ^2 p value	OR (95%CI)	p value
REALM			<0.001		
Low	55.9	44.1		1.63 (0.63-4.24)	0.316
Marginal	73.1	26.9		1.09 (0.55-2.16)	0.811
Adequate	82.7	17.3		Ref	Ref

Note: Multivariable analyses were adjusted for age, gender, race, education, smoking, exercise frequency, alcohol consumption, BMI, and chronic conditions, time since baseline interview (≤36 months, >36 months) and baseline physical function
